# Safety and Immunogenicity of H5N1 Influenza Vaccine Based on Baculovirus Surface Display System of *Bombyx mori*

**DOI:** 10.1371/journal.pone.0003933

**Published:** 2008-12-12

**Authors:** Rongzhong Jin, Zhengbing Lv, Qin Chen, Yanping Quan, Haihua Zhang, Si Li, Guogang Chen, Qingliang Zheng, Lairong Jin, Xiangfu Wu, Jianguo Chen, Yaozhou Zhang

**Affiliations:** 1 College of Life Sciences, Zhejiang University, Hangzhou, China; 2 Institute of Biochemistry, College of Life Sciences, Zhejiang Sci-Tech University, Hangzhou, China; 3 College of Life Sciences, Peking University, Beijing, China; University of Helsinki, Finland

## Abstract

Avian influenza virus (H5N1) has caused serious infections in human beings. This virus has the potential to emerge as a pandemic threat in humans. Effective vaccines against H5N1 virus are needed. A recombinant *Bombyx mori* baculovirus, Bmg64HA, was constructed for the expression of HA protein of H5N1 influenza virus displaying on the viral envelope surface. The HA protein accounted for approximately 3% of the total viral proteins in silkworm pupae infected with the recombinant virus. Using a series of separation and purification methods, pure Bmgp64HA virus was isolated from these silkworm pupae bioreactors. Aluminum hydroxide adjuvant was used for an H5N1 influenza vaccine. Immunization with this vaccine at doses of 2 mg/kg and 0.67 mg/kg was carried out to induce the production of neutralizing antibodies, which protected monkeys against influenza virus infection. At these doses, the vaccine induced 1:40 antibody titers in 50% and 67% of the monkeys, respectively. The results of safety evaluation indicated that the vaccine did not cause any toxicity at the dosage as large as 3.2 mg/kg in cynomolgus monkeys and 1.6 mg/kg in mice. The results of dose safety evaluation of vaccine indicated that the safe dose of the vaccine were higher than 0.375 mg/kg in rats and 3.2 mg/kg in cynomolgus monkeys. Our work showed the vaccine may be a candidate for a highly effective, cheap, and safe influenza vaccine for use in humans.

## Introduction

During the period of May through December of 1997, an outbreak of human influenza A (H5N1) infection in the Hong Kong of China gave the serious cause for concern [1]. At the time there was no indication whether human infections would remain linked to the outbreak of poultry infections or whether H5N1 virus would acquire the ability to be transmitted from person-to-person [2]. From January 1 to March 31, 2004, 12 patients were confirmed to infect H5N1 influenza virus in Thailand [3], Human infections with influenza A (H5N1) were identified in 10 patients in Vietnam in January 2004 [4]. Widespread vaccination is the preferred strategy for preventing or at least limiting potential pandemic influenza outbreaks. The most expeditious way to generate H5N1 vaccine was to use the licensed technology, such as inactivated [5] or attenuated viral vaccines [6]. However, there are several practical and scientific challenges to the development of H5N1 vaccines [7]. These include the high pathogenicity of wild-type H5N1 influenza viruses, reduced yields of candidate vaccine viruses in the embryos of fertilized hen's eggs compared to yields of human influenza viruses, limited manufacturing capacity, and poor immunogenicity of H5 HA. Despite these obstacles, several approaches have been used to generate candidate vaccines and a few have advanced to clinical trials [8]. Clinical trails have been completed for vaccines. That include inactivated viral vaccines based on H5N1 viruses isolated in 2004 [9], [10] and a recombinant H5 HA subunit vaccine based on the H5N1 virus HA gene isolated in 1997, expressed in a baculovirus vector [11]. A subunit H5N1 vaccine based on A/Vietnam/1203/04 H5N1 virus was developed through reverse genetics and was produced by Sanofi Pasteur. Its effectiveness was assessed in a randomized trial among healthy adults in the USA [12]. The addition of MF59 adjuvant substantially boosted immune responses to this vaccine [13], [14]. Hungarian investigators (Omnivest, Budapest) also reported promising results for an aluminum phosphate adjuvant whole-virion H5N1 vaccine. A single dose of the vaccine containing 30 µg of H5 antigen, induced seroconversions, as determined by haemagglutinin inhibition, in 18 (90%) of 20 recipients [15].

The baculovirus expression vector system (BEVS) was established early in the 1980s [16]. At present, two baculovirus systems have been extensively used: the AcNPV and the BmNPV systems [17]. Since then a variety of heterologous genes had been efficiently expressed in BEVS.

Recombination rescue technology employing linear viral DNA vectors greatly improves the efficiency of creating recombinant viruses. Using this method, Possee *et al* developed a linear AcNPV expression vector (BacPAK6) that had recombinant efficiency of over 80% [18]. To generate a recombinant strain of BmNPV virus, BmBacPAK6, we co-transfected *BmN* cells with BacPAK6 and BmNPV DNA, and used a homologous recombination method that increased the frequency of recombinant virus production to 100% [19]. Currently, we have successfully expressed several bioactive recombinant proteins using the BmBacPAK6 expression system and *B. mori* pupae as a bioreactor. In addition, we have carried out a large-scale expression and purification of recombinant hGM-CSF in *B. mori* pupae and developed a new approach to the oral administration of these recombinant proteins [20].

Lu *et al*. utilized the baculovirus display system to construct a recombinant baculovirus displaying HA of the avian influenza virus (AIV) on the viral surface, imparting hemagglutination activity to the recombinant virus. Intramuscular injection of the purified HA-displaying baculovirus into a mouse, stimulated the production of antibodies that inhibited hemagglutination activity and neutralized influenza viral infections [21]. In a subsequent study, Yang *et al.* concluded that the gp64 cytoplasmic domain (CTD) resulted in highly efficient HA incorporation into the baculovirus envelope [22]. Foot-and-mouth disease virus (FMDV) site A, which is crucial to FMDV infections, was fused with the gp64 and displayed on the surface of baculovirus by Tami *et al*. Immunization of the mice with the recombinant baculovirus expressing the gp64-site A fusion protein elicited positive ELISA results and high seroneutralizing titers (SNT) against FMDV [23]. To evaluate the baculovirus display system as a vaccine vehicle, Yoshida *et al*. generated a recombinant baculovirus (AcNPV-CSPsurf) system. That displayed the rodent malaria plasmodium berghei circumsporozoite protein (PbCSP) on the viral surface as a fusion protein with the baculovirus envelope glycoprotein gp64. Immunization with adjuvant-free AcNPV-CSPsurf virions induced high levels of antibodies against PbCSP and protected 60% of the mice against a sporozoite challenge [24].

We constructed recombinant baculovirus Bmgp64HA, which contained a gp64HA fusion gene under a control of a polyhedron promoter. The gp64HA fusion gene contained a synthetic HA fragment of H5N1 virus fused to the portions of the gp64 gene that encodes the signal peptide and the transmembrane domain, allowing display of highly expressed HA on the surface of the baculovirus envelope. The content of HA protein in the recombinant virus was 3% of the total baculovirus proteins. Evaluation of the vaccine in the mice and monkeys suggested that the vaccine was safe. Mice and monkeys immunized with this recombinant virus induced production of neutralizing antibodies, which provided protection against H5N1 infections.

## Materials and Methods

### Recombinant Virus

**Figure 1:**
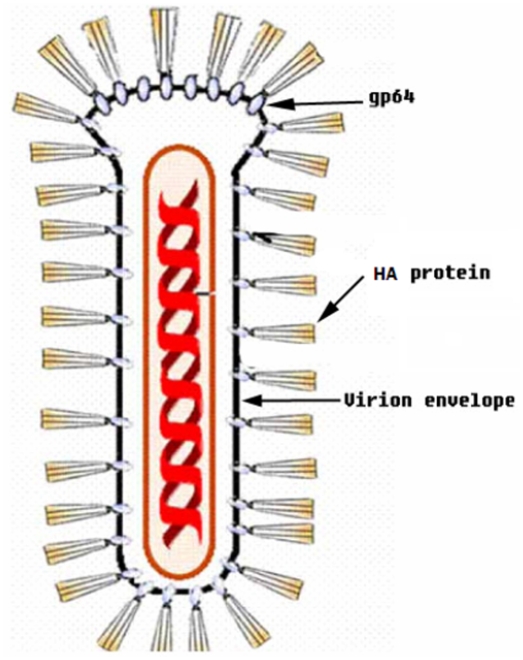
An ideograph of HA/gp64-fusion recombinant baculovirus

The recombinant baculovirus Bmgp64HA was kindly provided by Professor Chen J.G. of Peking University, as shown in Fig. 1. Based on the HA sequence of the Hangzhou strain of influenza virus A/Zhejiang/16/06(H5N1), we constructed a fusion gene containing a HA gene fragment without both signal peptide and transmembrane domain. The fragment was inserted the in-frame with a sequence encoding the *B.mori* baculovirus gp64 signal peptide and transmembrane domain. This fusion gene was used to generate the recombinant baculovirus Bmgp64HA via a homologous recombination.

### Separation and purification of the recombinant baculovirus

*BmN* cells were infected with Bmgp64HA at an MOI of 10 infection indexes to generate the amplified recombinant virus used to infect *B. mori* pupae by a pinprick inoculation at a density of 1×10^6^ pfu/strip. The pupae were collected 5 to 7 days after infection, homogenized, and centrifuged at 12,000 rpm for 30 min. The supernatant was collected and HA bioactivity was determined using the HA assay. The expression of the gp64HA fusion protein in the pupae was identified by both SDS-PAGE and western blotting.

The recombinant Bmgp64HA was separated and purified as described as below: (1) Pupae infected with Bmgp64HA virus were homogenized in 0.85% NaCl. The pupae homogenates were centrifuged in following steps at 3,000 rpm for 30 min, 6,000 rpm for 30 min, 12,000 rpm for 60 min, and 18,000 rpm for 60 min at 4°C. The supernatant was ultracentrifuged at 30,000 rpm for 40 min at 4°C to precipitate viral particles. The viral pellet was resuspended in a sterile solution (0.05 M PB, 0.5 M NaCl, 0.04%EDTA). To further purify the viral particles, the suspension was used in a sucrose density gradient ultracentrifugation. (2) Sucrose was dissolved in a solution containing 0.05 M PB, 0.5 M NaCl, and 0.04% EDTA. The density gradient was layered as follows: the first layer contained a sterile solution (0.05 M phosphate buffer (PB), 0.5 M NaCl, 0.04% EDTA); the second layer the sample; the third layer 20% sucrose; the fourth layer 30% sucrose; and the fifth layer the 52% sucrose solutions to fill the centrifuge bottle. After a 3-h centrifugation at 35,000 rpm, samples were collected in sterile tubes. The sucrose solution in the samples was removed by using a 750 kD hollow filter cartridge. (3) The sample bioactivity was determined using a hemagglutination (HA) assay. (4) The samples with high bioactivity were freeze-dried. That power was resuspended with Al(OH)_3_ adjuvant in 0.9% NaCl (V/V = 1:9). The prepared solution was used as H5N1 influenza vaccine.

### Vaccine protection against viral challenge in rhesus monkeys

The vaccine trial was approved by the Institutional Animal Care and Use Committee (IACUC) of Institute of Laboratory Animal Sciences, Chinese Academy of Medical Science (The approved No.: N-07-6001). The trial complied with the institutional guidelines of the animal welfare and husbandry for monkeys. Measures were taken to avoid or ameliorate the suffering of the monkeys, which include both anesthesia and euthanasia. The studies were performed in accord with the predetermined study design and objectives.

Twenty rhesus monkeys including 10 females and 10 males were 3 to 4 years old and weighed 3.5 to 5.0 kg. Beijing Xie'rxin Biological Resources Institute supplied the experimental animals that were maintained appropriately prior to viral challenge and under Biosafe P3 level conditions after challenge. Those monkeys were randomly divided into 5 groups, including 2 control groups of Al(OH)_3_ adjuvant control (CN1) and 0.9% NaCl solvent control (CN2). Those in the other groups were immunized with either a high-dose (TA), a medium-dose (TB), or a low-dose (TC) of the vaccine. There were no significant differences in the weights of animals in the above various groups.

The doses administered (1 ml each) contained either 2 mg/kg of the vaccine (TA); 0.67 mg/kg of the vaccine (TB); 0.22 mg/kg of the vaccine (TC); Al(OH)_3_ adjuvant in 0.9% NaCl (CN1); or 0.9% NaCl (CN2). Each animal received 2 cervical 0.5 ml subcutaneous injections, simulating the clinical method of administration. Those animals were immunized once every 2 weeks; the immunization frequency was determined in accord with neutralizing antibody titer levels. When the neutralizing antibody titers reached their highest levels the animals were challenged with virus (1×10^6^ pfu in a 1 ml solution) by a nasal drip.

The antibody titers were determined after the first immunization. Those animals were challenged with the influenza virus when antibody titers reached their highest levels and the animals were euthanized on Day 7 and Day 14 post-challenges. The viral strain used in challenges was A/tiger/harbin/01/2002(H5N1). The vaccine protection was evaluated by examining animals for clinical symptoms, body temperature, blood biochemistry, blood routine, and histopathological lesions. Those animals were also assayed for viral genomes by RT-PCR, and neutralizing antibodies, ELISA methods, viral isolations. The virus in throat swabs on Days 2, 5, 7, 14 post-challenge and lung tissues in necropsy was detected by RT-PCR. Serum samples were collected and inactivated at 56°C for 30 min after 2 weeks of a post vaccination and on Days 2, 5, 7, 14 post-challenges. The mixture of sera by a 2-fold dilution and the standard virus was added to MDCK cells in the microplate. Cytopathic effects were observed after 3–4 days of culture in 37°C. The titers of neutralizing antibody were recorded. The antibody titers were measured by ELISAs with the inactive influenza virus as the ELISA antigens.

### Safety evaluation of vaccine in mice and cynomolgus monkeys

The Experimental Animals Center of Zhejiang province supplied 100 males and 100 females ICR mice at the weights of 19 to 21 g. The mice were inoculated with the high-dose, TA of 16 mg/kg, the medium-dose, TB of 8 mg/kg or the low-dose, TC of 4 mg/kg of the vaccine. The solvent control group was injected with a 5 ml/kg dose of Al(OH)_3_ adjuvant in 0.9% NaCl (V/V = 1:9), and the negative control group was injected with a 5 ml/kg dose of 0.9% sodium chloride. Dosages were prepared in a solvent by isochoric suspension for use in subcutaneous injections. The effect of vaccination on the nervous system was surveyed.

Suzhou Xishan Zhongke Experimental Animals Ltd Co. supplied 10 males and 10 females, 3 to 4 years old, cynomolgus monkeys at 2.5 to 3.0 kg each. The monkeys were inoculated with the high-dose, TA of 3.2 mg/kg, the medium-dose, TB of 1.6 mg/kg or the low-dose, TC of 0.8 mg/kg of the vaccine. The solvent control group was injected with a 1 ml/kg dose of Al(OH)_3_ adjuvant in 0.9% NaCl (V/V = 1:9), and the negative control group was injected with a 1 ml/kg dose of 0.9% NaCl. The effect of vaccination on cardiovascular systemrespiratory system was surveyed.

### Dose safety evaluation in rats and cynomolgus monkeys

The SD rats was vaccinated 8 times every 10 days by a subcutaneous injection. Three of 5 groups were vaccinated with 9.4, 1.88, 0.375 mg/kg/injection of the vaccine, respectively. Two of the 5 groups were injected with a solvent control and 0.9%NaCl 1 ml/kg/injection,respectively.

Cynomolgus monkeys were vaccinated 8 times every 10 days by a subcutaneous injection. Three of 5 groups were vaccinated with 3.2, 1.6, 0.8 mg/kg/injection of the vaccine, respectively. Two of the 5 group were injected with a solvent control and 0.9%NaCl 1 ml/kg/injection,respectively. Toxic reactions of the vaccine to rats and cynomolgus monkeys were monitored.

## Results

### Expression by Bmgp64HA in Bombyx mori pupae

**Figure 2:**
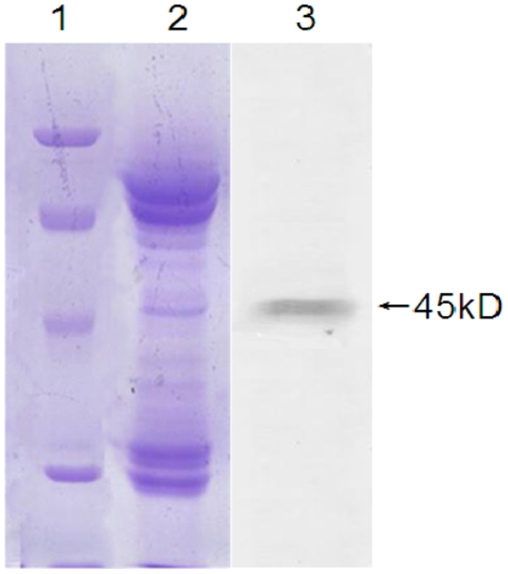
The expression of a 45 kD HA protein of influenza by recombinant Bmgp64HA. The recombinant baculovirus was purified from pupae by four steps centrifugation. Lane M. Protein molecular weight marker. Lane 1. The purified recombinant baculovirus. Lane 3. HA protein expression detected by western blotting.

**Figure 3:**
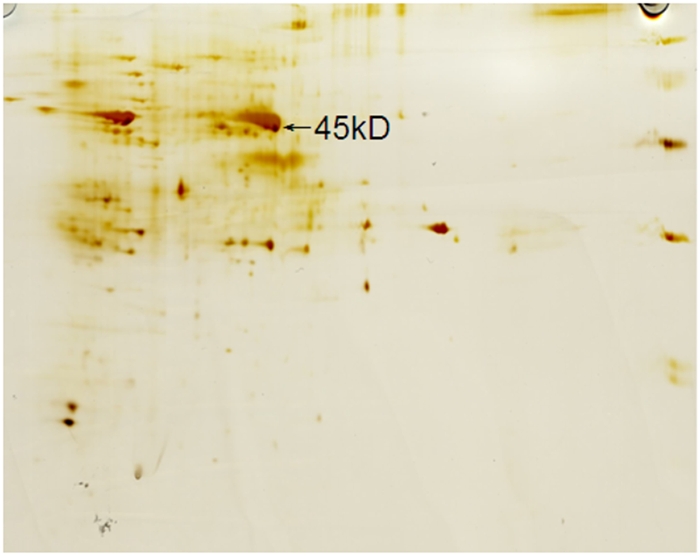
The ecombinant baculovirus Bmgp64HA was detected by 2-D electrophoresis. 45 kD HA protein was expressed in recombinant baculovirus.

Both SDS-PAGE and western blotting analyses revealed a 45 kD gp64HA fusion protein was expressed in the pupae (Fig. 2). The proteins of 270 µg purified from recombinant baculovirus were separated by a 2-D electrophoresis. The silver staining of the 2-D gel (Fig. 3) followed by MALDI-TOF-TOF analysis revealed the 45 kD gp64HA protein. The gp64HA fusion protein was approximately 3% of the total protein of Bmgp64HA determined by a TLC scanner, (CS-9301PC, Shimadzu). The yield rate of recombinant virus was 1 mg/*B. mori* pupae.

### Determination of antibody titers induced by vaccine in rhesus monkeys

Antibody titers were determined by ELISA after 2 weeks of post immunization. After the initial immunization, the antibody titers of one monkey in the high-dose (TA) group and one monkey in the medium-dose (TB) group were observed as high as 1:20; the antibody titers of 2 monkeys in the low-dose (TC) group were also 1:20; the rest of the monkeys were seronegative.

After the first booster immunization in the TA group, the antibody titer of one previously seronegative monkey (No.031275) was 1:80, and the antibody titer of another previously seronegative monkey (No. 031869) was 1:20; the antibody titer of one monkey (No. 040152) changed from1:20 to 1:40. In the TB group, the antibody titer of the previously seronegative monkey (No.040193) was 1:80 after the first booster immunization and the antibody titer of another monkey (No.040278) increased to1:80 while the other monkeys in the TB group, remained seronegative. In the TC group, the antibody titer of 2 monkeys changed from1:20 to 1:40. The antibody titers of other two monkeys in the TC group were 1:20, 1:80, respectively.

After the second booster immunization, most of monkeys maintained neutralizing antibody titers at their previous levels. However, 2 previously seronegative monkeys in the medium-dose group produced antibody titers of 1:20 and 1:40, respectively. The antibody titers of the rhesus monkeys as measured by ELISA were shown in Table 1.

**Table 1:** The antibody titers of the rhesus monkeys were measured by ELISA.

Group	First vaccination		Second vaccination				Third vaccination			
Antibody titer	0	1:20	0	1:20	1:40	1:80	0	1:20	1:40	1:80
TA	3/4	1/4	1/4	1/4	1/4	1/4	1/4	1/4	1/4	1/4
TB	3/4	1/4	2/4	-	1/4	1/4	-	1/4	3/4	-
TC	2/4	2/4	-	1/4	2/4	1/4	-	1/4	1/4	2/4
CN	4/4	-	4/4	-	-	-	4/4	-	-	-

The numbers of seropositive monkeys were shown.

Each of the groups had 4 monkeys.

### Protective immunity induced by vaccine in rhesus monkeys

Neutralizing antibody titers reached their highest levels after animals were inoculated with the vaccine for 3 times. No abnormal clinical responses were observed during the virus challenge, and body temperatures and the indexes of blood biochemistry and blood routine remained in the normal range after the viral challenge.

A histopathological examination revealed the 2 monkeys had a severe interstitial pneumonia and other 2 monkeys had a moderate to severe interstitial pneumonia in the control group. All monkeys in TA group had a mild interstitial pneumonia, and 4 monkeys in TB group had a mild to moderate interstitial pneumonia. All of the animals in both TA and TB groups were protected from the interstitial pneumonia by the vaccine. In the TC group, one monkey had a mild to moderate interstitial pneumonia, and other 3 monkeys had a moderate to severe interstitial pneumonia.

### RT-PCR results in rhesus monkeys after viral challenge

RT-PCR was used to detect H5N1 virus in tissues of rhesus monkeys after the viral challenge. Using throat swab samples from two monkeys, virus was detected in each of the dose groups (TA, TB, TC) after 2 days of the viral challenge. One monkey in the control group was observed positive. After 5 days of the viral challenge, virus was not detected in any of the monkeys. Lungs of two monkeys in the control group were observed viral positive after 7 days after the viral challenge; and one monkey in each vaccine dose group was also positive. Fourteen days of the viral challenge, all of lung tissues were tested viral negative. These results are shown in Table 2.

**Table 2:** Summary of RT-PCR results for viral detection in throat swab samples of monkeys following viral challenge after immunization with the vaccine.

Group	2 d	5 d	7 d	14 d
TA	2/4	0/4	0/4	0/4
TB	2/4	0/4	0/4	0/4
TC	2/4	0/4	1/4	0/4
CN	1/4	0/4	2/4	0/4

Each of the groups had 4 monkeys.

### Neutralizing antibody titers in rhesus monkeys after viral challenge

In vaccinated rhesus monkeys, neutralizing antibodies were determined at Days 0, 2, 5, 7, and 14 after the viral challenge. The results were shown in Table 3. The neutralizing antibody levels declined transiently after the viral challenge. In 6 of the monkeys, the neutralizing antibody titer had decreased after 2 days of the viral challenge. However the neutralizing antibody titers of 1:8 or 1:4 were observed in two monkeys that were previously seronegative after 7 days of the viral challenge. The neutralizing antibody titers reached 1:8 in 7 of 12 monkeys, 1:4 in 3of 12 monkeys, and negative in 2 of 12 monkeys. By the contrast, the titer of 1:4 was observed in 2 of 4 monkeys in the control group.

**Table 3:** Summary of neutralizing antibody titers of monkey following viral challenge after immunization.

		CN	TA	TB	*TC*
**0 day**	Seropositive number		2/4	3/4	2/4
	Titer range		1:8	1:4–1:8	1:4
**2 day**	Seropositive number		2/4	1/4	1/4
	Titer range		1:4	1:4	1:4
**5 day**	Seropositive number		1/4	0/4	1/4
	Titer range		1:4		1:4
**7 day**	Seropositive number	2/4	4/4	3/4	3/4
	Titer range	1:4	1:4	1:4–1:8	1:4–1:8
**14 day**	Seropositive number	2/4	2/4	2/4	2/4
	Titer range	1:8	1:16	1:8–1:16	1:8

Each of the groups had 4 monkeys.

### Virus isolation after challenge in rhesus monkeys

After the viral challenge, viral genomes were detected by means of RT-PCR in throat swab samples of one monkey in the TB group. No virus was detected in samples of the monkeys in either TA or TC groups after 2 days of the viral challenge while all of the monkeys in the control group were viral positive. No virus was detected in the monkeys of the control group after 5, 7, or 14 days of the viral challenge. After 7 days of the viral challenge, virus was isolated from the lung tissue of 1 monkey in the TC group and 1 monkey from the control group, respectively.

### Safety evaluation of vaccine in mice and cynomolgus monkeys

The results of the general behavior, locomotor activity, subthreshold hypnotic dosage, sodium pentobarbital cooperative experiment and rotating coordination function assay showed that no singnificant effects on the nervous system of the mice was observed after administration of the influenza vaccine by the subcutaneous injection with 16, 8, 4 mg/kg, solvent 5 ml/kg.

Examinations on the cardiovascular and respiratory system in cynomolgus monkey showed that each of indexes was not influenced markedly by inoculation of influenza vaccine in 3.2, 1.6,0.8 mg/kg and solvent 1 ml/kg dose. No significant effects on the monkeys were observed in the blood pressure, respiratory frequency and amplitude electrocardio parameter after the vaccination (*P*>0.05). Also the vaccine showed no apparent effect on body temperature, and means temperature on each time point after immunization (*P*>0.05) as compared to the control group. The similar results were observed in the monkeys, which the vaccine had no effects on the monkey's body temperature, cardiovascular and respiratory system.

### Dose safety evaluation of vaccine in rats and cynomolgus monkeys

The SD rats was vaccinated eight times by the subcutaneous injection. Granuloma nodules observed at the injection site gradually were diminished after discontinuation of the inoculations. There was no obvious change in the blood parameters, organ weight, liver index, spleen weight, and other parameters. The toxic effect was observed in spleen; however, the effects were reversible. The toxic reactions were observed after inoculation with vaccine dose of 1.88 mg/kg, while no toxic reactions observed after the inoculation with a vaccine dose of 0.375 mg/kg. The primary toxic reactions of scabs and red patches at the injection site were observed in the monkeys inoculated with the vaccine of a a 640-fold dose refered to the human clinical dose. The reactions were probably caused by the deposition of vaccine protein and aluminum hydroxide adjuvant in the subcutaneous tissue. No obvious toxic reaction was observed in other organs of the monkeys.

**Figure 4:**
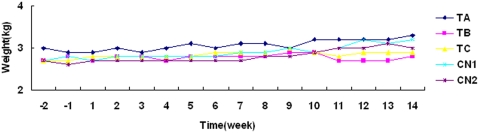
The weight change in a long-term experiment in monkeys immunized with the influenza vaccine. ‘−1’ refers to 1 week before the vaccine immunization. Vaccine immunizations occurred during weeks 1 to 10. Weeks ‘11 to 14’ refered to the time post-immunization.

Cynomolgus monkeys were inoculated with the vaccine by subcutaneous injection 8 times with a dose of 3.2, 1.6, or 0.8 mg/kg in solution (1 ml/kg), or with the solution alone. No long-term weight change was observed in the monkeys immunized with the vaccine (Fig. 4). The safe dose of the vaccine was observed to be higher than 3.2 mg/kg.

**Figure 5:**
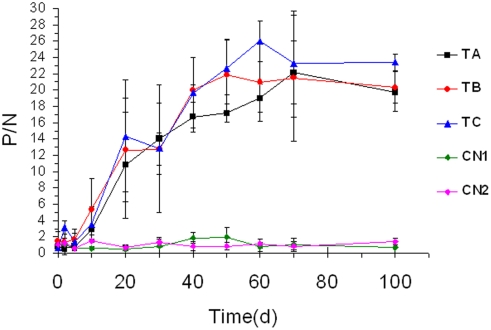
Anti-HA antibody titers of the vaccinated monkeys in a long-term experiment.

Anti-AIV HA antibody titers of the vaccinated monkeys were maintained for 3 months and increased gradually, reaching their highest plateau after the fifth immunization, which no any significant changes were observed between anti-HA antibody titers and the vaccine dose administered to the monkeys (Fig. 5). One month after the eighth immunization, serum HA antibody titers in the vaccinated monkeys were still maintained at the highest level. HA antibody titers of the monkeys in the solvent control and 0.9% NaCl control groups were not affected by the vaccination.

## Discussion

Both Influenza A and B viruses spread in human nearly every winter in temperate regions of both the Northern and Southern hemispheres and throughout the year in tropical climates [25]. The impact of annual influenza epidemics are difficult to assess due to its co-circulation with other agents that have adverse respiratory impacts. The nonspecific illnesses are also associated with influenza infection, the available estimates of the impact of influenza are nevertheless impressive. As reported 6,200 to 29,000 people died of each of the epidemics occurred between 1975 and 1976, and 1984 and 1990 [26] in England and Wales, and average of 51,203 people died annually during influenza outbreaks in the United States between 1976 and 1999 [27]. Three percent of the local population died during an influenza outbreak in Madagascar in 2002 [28]. Large increases in absenteeism from schools and works, and increased hospital admissions and consultations with general practitioners occur during influenza outbreaks.

A vaccine providing 40 to 50% protection against the human H5N1 influenza was developed in 2007 [29]. Additionally, a new avian influenza vaccine, providing up to 70% protection of the human population, was reported [30]. In comparison, seasonal vaccinations provide protection against influenza to approximately 90% of healthy young people [31]. The clinically effective dose of avian influenza vaccine for human is typically 10 to 90 µg [29], [30].

There are three types of influenza vaccines including whole virus vaccines, split vaccines, and subunit vaccines. Because the vaccine production requires viral replication, which is risky in that virus from the production facility could escape and possibly disseminate into the human population. Alternatively, the use of chick embryos to generate vaccines against H5 viruses could be challenging because influenza viruses grown for vaccine production are lethal to the embryos [32]. To date, the various technologies used to produce vaccines showed to be inefficient [33], [34]. The use of genetic engineering technologies to create vaccines rather than conventional methods of using inactivated live virus may increase production efficiencies and decrease the possibility of the viral spreading into the population.

Significant efforts have been made toward developing effective human influenza vaccines [35], [36]. Mary *et al*. developed a replication-incompetent, human adenoviral-vector-based, hemagglutinin subtype H5 influenza vaccine (HAd-H5HA) that induces both humoral and cell-mediated immune responses against avian H5N1 influenza viruses isolated from people [37]. Immunization with the HAd-H5HA vaccine induced the effective protection in mice from the disease, death, and primary viral replication (*P*<0.01) caused by antigenically distinct strains of H5N1 influenza viruses [38], [39].

Recently, a novel eukaryotic surface display system based on a deep understanding of the construction and function of the baculovirus genome has been developed. John *et al*. used an AcNPV expression system that expressed H5 HA to immunize human [11]. Their results suggested that baculovirus-expressed H5 HA induces the production of neutralizing antibodies in human. An innovative monitoring assay for the recombinant HA expression and a rapid purification process has also been reported [40]. Researchers utilized the AcNPV system to express H5, H7, and H9 subtype hemagglutinins as vaccines to protect against lethal influenza infections. Lu *et al.* used AcNPV a surface display expression of the hemagglutinin of H5N1 influenza virus to immunize mice. The intramuscular injection of the purified HA-displaying AcNPV into mice induced the production of neutralizing antibodies [21]. Baculovirus infected insect cells release budding virus from the cells into the intercellular space.

The gp64, the most abundant glycoprotein in type I budding baculovirus including AcMNPV and BmNPV and forms trimers through disulfide crosslinking on the surface of the baculovirus, can mediate the viral infection of cells [41]. The BmNPV gp64 signal peptide is located in the N-terminal region and its transmembrane domain in the C-terminal of the protein,. By fusing exogenous peptides to these portions of the gp64 protein, they can be displayed on the viral envelope surface. At present, peptides derived from several eukaryotic proteins have been expressed on the baculovirus surface. These are limited to the AcNPV expression systems of the *Sf* cell line, but it is not yet suitable for a large scale of manufacturing.

Our laboratory has developed *B. mori* pupae as a bioreactor system for highly efficient expression of hGM-CSF [20]. We have also constructed a linearized BmNPV expression system, BmBacPAK6 [42] expressing a fusion gene including the HA gene of H5N1 (Hangzhou strain) [43] which is inserted the in-frame with the sequence that encodes the BmNPV gp64 signal peptide and transmembrane domain to create baculovirus Bmgp64HA. The fusion gene expresses a high level of the 45 kD gp64HA fusion protein. We were able to isolate pure Bmgp64HA from infected pupae. The yield was 1 mg per *B. mori* pupae. The HA content of the total viral protein was measured at approximately 3%. This system may provide a fundamental platform for the large-scale vaccine production.

We used the recombinant Bmgp64HA to vaccinate rhesus monkeys against H5N1 influenza virus. The results showed that in the rhesus monkeys, inoculated with 2 mg/kg, 0.67 mg/kg, 0.22 mg/kg of vaccine after three vaccinations, could induce the production of over 1:40 antibody titers in 50%, 67%, or 67% of the monkeys, respectively.

We have successfully constructed a baculovirus surface display system. One example of this surface display system is the recombinant Bmgp64HA virus, which efficiently displays the H5N1 influenza virus HA on the baculovirus envelope surface. This recombinant virus can be used as a safe and effective vaccine against H5N1 infection in monkeys. Our recombinant virus provides a highly efficient, cost effective, and safe influenza vaccine. And our improved baculovirus display system could be used for the production of other types of vaccines.
